# Highly Differentiated, Resting Gn-Specific Memory CD8^+^ T Cells Persist Years after Infection by Andes Hantavirus

**DOI:** 10.1371/journal.ppat.1000779

**Published:** 2010-02-19

**Authors:** Tobias Manigold, Andrés Mori, Rebecca Graumann, Elena Llop, Valeska Simon, Marcela Ferrés, Francisca Valdivieso, Constanza Castillo, Brian Hjelle, Pablo Vial

**Affiliations:** 1 Institute of Science, Medical School, Clínica Alemana Universidad del Desarrollo, Santiago, Chile; 2 Facultad de Medicina, Universidad de Chile, Santiago, Chile; 3 Facultad de Medicina, Pontificia Universidad Católica de Chile, Santiago, Chile; 4 Universidad de la Frontera, Temuco, Chile; 5 University of New Mexico, Albuquerque, New Mexico, United States of America; University of Washington, United States of America

## Abstract

In man, infection with South American Andes virus (ANDV) causes hantavirus cardiopulmonary syndrome (HCPS). HCPS due to ANDV is endemic in Southern Chile and much of Argentina and increasing numbers of cases are reported all over South America. A case-fatality rate of about 36% together with the absence of successful antiviral therapies urge the development of a vaccine. Although T-cell responses were shown to be critically involved in immunity to hantaviruses in mouse models, no data are available on the magnitude, specificity and longevity of ANDV-specific memory T-cell responses in patients. Using sets of overlapping peptides in IFN-γ ELISPOT assays, we herein show in 78 Chilean convalescent patients that Gn-derived epitopes were immunodominant as compared to those from the N- and Gc-proteins. Furthermore, while the relative contribution of the N-specific response significantly declined over time, Gn-specific responses remained readily detectable *ex vivo* up to 13 years after the acute infection. Tetramer analysis further showed that up to 16.8% of all circulating CD3^+^CD8^+^ T cells were specific for the single HLA-B*3501-restricted epitope Gn_465–473_ years after the acute infection. Remarkably, Gn_465–473_–specific cells readily secreted IFN-γ, granzyme B and TNF-α but not IL-2 upon stimulation and showed a ‘revertant’ CD45RA^+^CD27^−^CD28^−^CCR7^−^CD127^−^ effector memory phenotype, thereby resembling a phenotype seen in other latent virus infections. Most intriguingly, titers of neutralizing antibodies increased over time in 10/17 individuals months to years after the acute infection and independently of whether they were residents of endemic areas or not. Thus, our data suggest intrinsic, latent antigenic stimulation of Gn-specific T-cells. However, it remains a major task for future studies to proof this hypothesis by determination of viral antigen in convalescent patients. Furthermore, it remains to be seen whether Gn-specific T cells are critical for viral control and protective immunity. If so, Gn-derived immunodominant epitopes could be of high value for future ANDV vaccines.

## Author Summary

In man, hantavirus cardiopulmonary syndrome (HCPS) caused by Andes Virus (ANDV) is endemic in the Southern cone of Chile and Argentina but cases of HCPS are being increasingly reported all over South America since 1995. HCPS is characterized by fulminant pulmonary edema which progresses to shock and death in about 36% of patients with HCPS. Nevertheless, to date, neither antiviral treatments nor vaccines inducing neutralizing antibodies (NAb) have proven effective against HCPS-causing hantaviruses. We set out for the first study on human cellular immunity towards ANDV in 78 convalescent survivors of ANDV infection. We found that Gn-specific responses were predominant as compared to N- and Gc-specific responses, even up to 13 years after the infection. Surprisingly, most of the Gn-specific responses were restricted to two neighboring epitopes within the Gn carboxyterminus. Interestingly, among HLA-B*3501^+^ patients, Gn_465−473_-specific CD8^+^ T-cells showed highly differentiated but resting phenotype and functions. It remains to be seen in future studies whether the immunodominace of Gn-specific T-cells is crucial for protective immunity. Most intriguingly, titers of neutralizing antibodies increased in 10/17 individuals months to years after the acute infection and independently of whether they were residents of endemic areas or not. Thus, our data suggest viral persistence or latency in part of ANDV-convalescent patients. However, it remains a major task for future studies to proof the concept of latent/persistent human ANDV infection by the determination of viral antigen in convalescent patients.

## Introduction

The family *Bunyaviridae* is comprised of five genera of tri-segmented negative-stranded RNA viruses, which are responsible for a considerable burden of zoonotic disease in man. While most are tick- or mosquito-borne, members of the genus *Hantavirus* are transmitted from chronically- and asymptomatically-infected rodents to humans via aerosols, which may derive from urine, feces or saliva. Globally hantaviruses may cause as many as 200,000 cases of human disease per year. In man, two clinical conditions may arise: hemorrhagic fever with renal syndrome, caused by the Asian and European strains (e.g. Hantaan, HTNV and Puumala, PUUV) or hantavirus cardiopulmonary syndrome (HCPS), which is caused by Sin Nombre virus (SNV) and Andes virus (ANDV), among others in the Americas. HCPS is an emerging infectious disease in North- and South America [1]-[5] and, currently, Chile represents among the most endemic regions for HCPS with more than 580 cases since 1995 [6].

As for ANDV, transmission to man is followed by infection of lung endothelial cells and, after an incubation period of 7 to 39 days [7], the development of a vascular leakage syndrome, eventually leading to massive pulmonary edema, shock and, in many cases, death. The high case-fatality ratio (mean 36%), the absence of a proven antiviral treatment or a vaccine, their mode of transmission and their potential use as weapons for bioterrorism, have rendered HCPS-causing hantaviruses Category A pathogens within NIAID's biodefense program [8]. Importantly, ANDV is the only hantavirus for which person-to-person transmission has been repeatedly documented [9]–[11].

The hantavirus virion contains a lipid-bilayer envelope into which both constituents, the Gn and Gc antigens of the heteromeric glycoprotein, are inserted via transmembrane domains. In the viral core, there are three nucleocapids each consisting of the RNA-binding N or nucleocapsid protein in complex with one of the genomic RNAs. These mRNAs encode the RNA-dependent RNA polymerase or L protein on the large or L segment (2153aa), the Gn (650aa) and Gc (490aa) glycoproteins on the middle or M segment, and the N protein (430aa) on the S segment [12].

Currently, there is a big discrepancy regarding the role of T cells in either pathogenesis or immunity of hantavirus infections. On one hand some studies in SNV-infected patients describe a correlation between the severity of HCPS and either the frequency of SNV-specific CD8^+^ T-cells [13] or the HLA-B35 haplotype [14], suggesting a T-cell driven pathogenesis of HCPS. On the other hand, several early reports highlight the importance of lymphocytes for immunity of mice towards hantaviruses, such as HTNV [15]–[17]. Likewise, clearance of HTNV in newborn mice was dependent on TNF-α production and cytotoxic activity of specific CD8^+^ T cells [18]. In addition, (HTNV-) N-protein-specific memory T-cells conferred partial protection and cross-protection towards N-expressing vaccinia virus [19] or hantaviruses [20] in mice and Syrian hamster [21],[22], respectively. In line with these findings we have recently reported that clearance of ANDV-RNA from peripheral blood cells of a patient was closely related to the appearance of cytotoxic CD8^+^ T cells about two months after the acute infection [23]. This observation together with the finding that we were unable to detect memory T-cells in many of the survivors of ANDV-induced HCPS (see below) led us to the hypothesis that T-cells may be crucial for protection and immunity towards ANDV rather than the pathogenesis in ANDV-infected patients. Concisely, knowledge of targeted epitopes and functional properties of ANDV-specific T-cells in ANDV-survivors may be important for both future studies in acutely ill patients and possibly for vaccine development.

Despite its importance, the knowledge of the human cellular immune response to hantaviruses is limited. To date, only few studies have assessed SNV-, HTNV- or PUUV- specific T cells [13], [24]–[28] in rather small patient cohorts and based on individual *in-silico*-predicted peptides and/or T-cell lines that had been expanded *in vitro*. Thus, the overall magnitude of human T-cell responses *in vivo* and the epitope-hierarchy within the memory T-cell pool in convalescent patients remains uncertain. Also, phenotype, effector functions and longevity of specific memory T-cells in humans remain to be elucidated. Greater knowledge of these matters would likely to be of high value to potential vaccine developers.

In an effort to gain insight into human cellular immunity to ANDV and to establish an immuno-hierarchy among ANDV-antigens, we carried out a study of the viral protein-specific T cell responses in 78 Chilean patients with past ANDV-infection. Our findings on the immuno-hierarchy among major structural hantavirus proteins and the frequencies as well as the functional features of CD8^+^ memory T cells may be of special interest for vaccine development since all attempts to induce long-lasting neutralizing humoral immunity have been unsuccessful so far [29].

## Results

### Gn of ANDV is highly immunogenic

In order to quantify circulating ANDV-specific T-cells *ex vivo* and to determine the immunodominant epitopes of ANDV, we first challenged PBMC of 78 Chilean convalescent patients (between 4 months and 13.2 years after hospitalization due to infection) with 310 overlapping peptides (distributed in 13 pools) spanning the entire N- and Gn/Gc precursor proteins [30] in IFN-γ ELISPOT assays.

**Figure 1:**
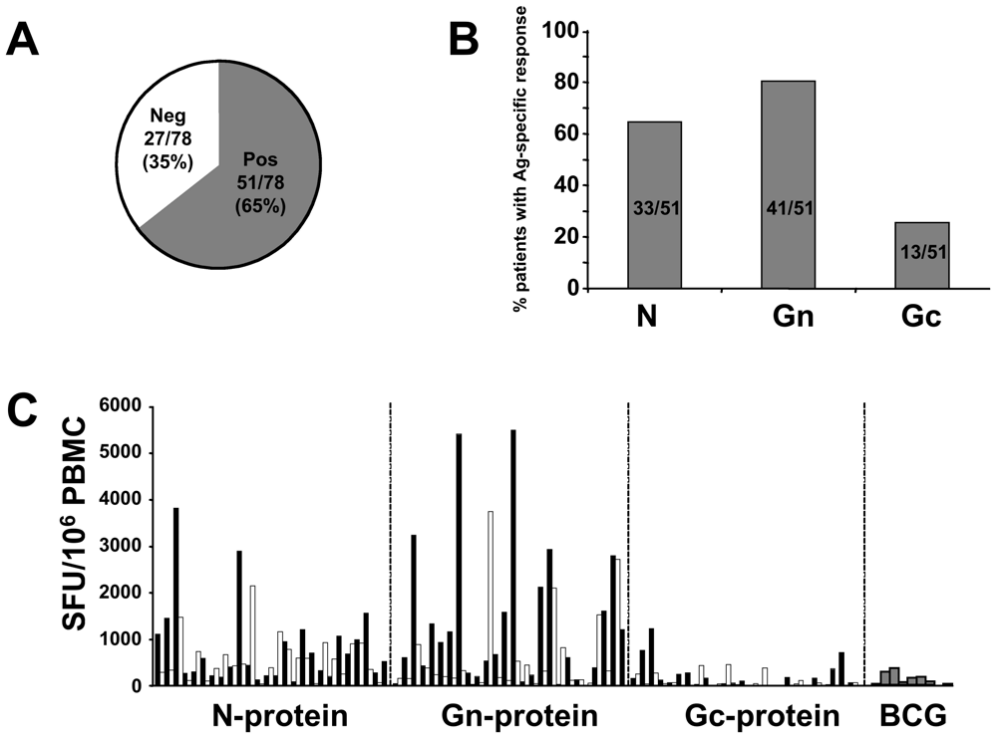
Distribution of ANDV-specific memory T-cell responses. (**A**) Summary of the determination of ANDV-specific T cells *ex vivo* IFN-γ ELISPOT assay in a total of 78 ANDV-convalescent patients. According to the criteria for positivity (see material and methods) only 51/78 (65%) displayed a positive response. Alternating black and white bar graphs (one bar represents one individual) are used for better visibility. (**B**) Differential recognition of ANDV antigens among the 51 responsive patients, indicating the relative immunodominance of Gn- as compared to N- and Gc-protein. (**C**) Individual T-cell responses to the different ANDV-antigens and BCG. Triplicates of PBMC of each patient were challenged in a 38-hour IFN-γ ELISPOT by a total of 13 pools of overlapping peptides, spanning the entire N- (aa 1–430), Gn- (aa 1–650) and Gc- (aa 641–1140) protein of Chilean ANDV. Most patients showed significant responses towards the Gn-antigen (aa 1–650). Each bar represents the sum of mean triplicate responses towards the peptides representing a given antigen. Depiction of standard-deviations was avoided to facilitate the visibility of results. The overall mean response to ANDV was 1805 SFU/10^6^ PBMC.

Based on the criteria we used to score a sample as “positive” (see Material and Methods), 51 (66%) of the 78 patients showed significant responses against epitopes of at least one of the three viral antigens (**Fig. 1A**). Among these patients, 33/51 (65%) showed significant responses against epitopes of the N-protein, while 13/51 (25%) showed Gc-specific T-cells (**Fig. 1B**). However, 80% (41/51) of the positive patients launched significant responses towards Gn-derived epitopes. Moreover, while mean responses among the 51 individuals reached the sum of 1809 Spot Forming Units (SFU)/10^6^PBMC when considering all viral antigens, Gn-specific responses accounted for more than half of the total response, at 973 SFU/10^6^PBMC (**Fig. 1C**) as compared to 697 and 139 SFU/10^6^ PBMC for N- and Gc-epitopes, respectively. Since all patients were BCG-vaccinated twice during childhood, we determined BCG-specific T cells (n = 10), resulting in a mean of 162 SFU/10^6^ PBMC. Thus, Gn is the immunodominant antigen in ANDV-convalescent individuals.

### Relative stability of the Gn- and Gc-specific memory T-cell pool

**Figure 2:**
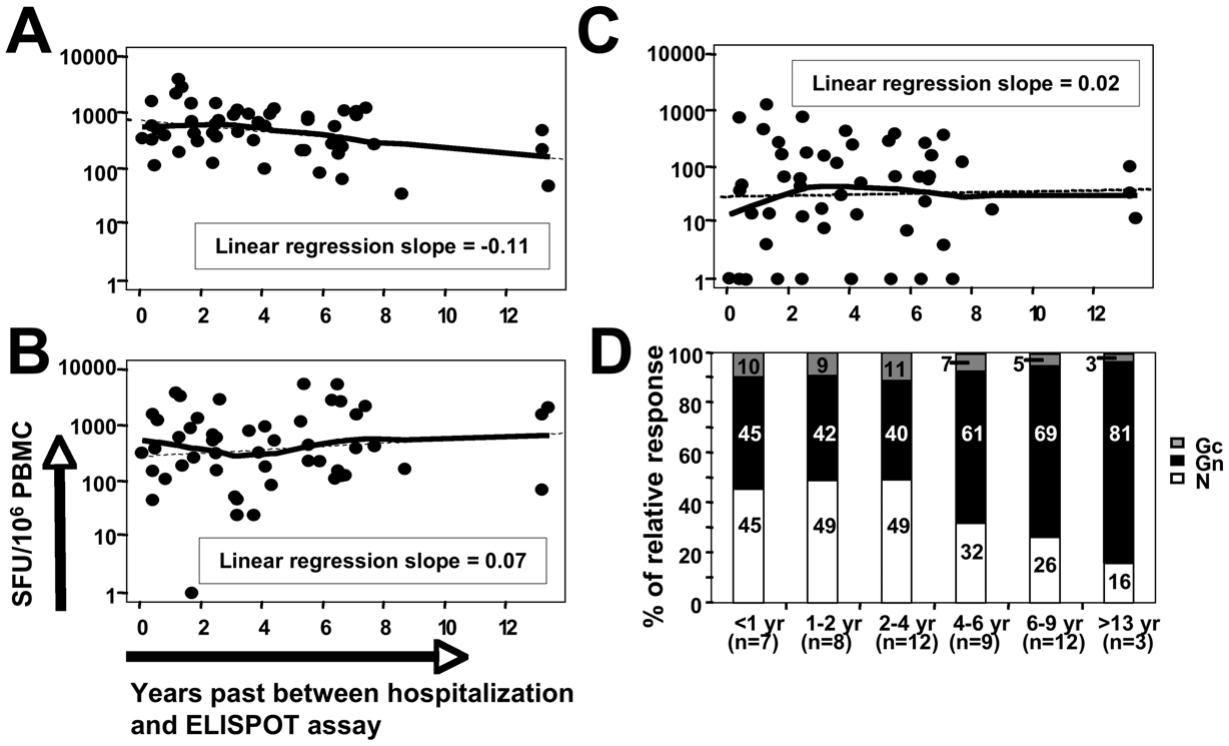
Relative contributions of antigen-specific memory T-cell responses over time. Association between N- (**A**), Gn- (**B**) and Gc-specific (**C**) memory T-cell responses and the time past between hospitalization and enrollment of a given patient. (**D**) Mean of antigen-specific contributions to absolute overall T-cell responses (defined as 100%, respectively) among 51 patients with positive T-cell responses and segregated into six groups according to the time past since hospitalization due to ANDV-infection. The numbers within the bar graphs indicate the relative percentages of N-, Gn- and Gc-specific responses, respectively.

We next asked whether differences in the longevity of each of the specific T-cell categories could account for the relative immunodominance of Gn among ANDV antigens, e.g., whether Gn-specific cells might persist longer than did cells responsive to the other antigens. We therefore considered the numbers of circulating N-, Gn- and Gc-specific T cells of each patient in relation to the time between the patient's hospital admission due to HCPS and the timepoint at which T cells were applied to ELISPOT assays (**Fig. 2A–C**). Although this approach does not allow to draw conclusions on the slope of antigen-specific responses at the level of a given individual, it is possible to directly compare the different antigen-specific responses within the overall cohort over time. Similar approaches have been previously performed on a cohort of smallpox vaccinees in order to estimate longevity and half-life of cellular and humoral memory responses [31].

Interestingly, only the N-specific response (**Fig. 2A**) exhibited a negative and significantly descending linear regression slope over time (r = −0.11, p<0.05), as when compared to Gn- and Gc-specific responses (**Fig. 2B, C**) (r = 0.07 and r = 0.02, respectively). We next segregated the 51 patients with positive T-cell responses into six groups according to the time past since hospitalization due to ANDV-infection (<1, 1–2, 2–4, 4–6, 6–9 and >13 years, respectively). As can be seen in Figure 2D, up to four years after infection N-specific responses were predominant, whereas afterwards Gn-specific relative contributions to the overall T-cell response increased from approximately 40% to more than 80% (**Fig. 2D**). Taken together, these data suggest that Gn- and Gc-specific T-cell responses are more stably maintained as compared to N-specific responses. However, in light of the limited value of the cross-sectional data available to us, future prospective studies assessing individual T-cell responses from the acute to the convalescent phase in individual patients would be needed to determine the absolute half-life of the N-, Gn- and Gc-specific T-cell responses.

### Immunodominance of Gn_461–475_ within the Gn carboxyl-terminus

**Figure 3:**
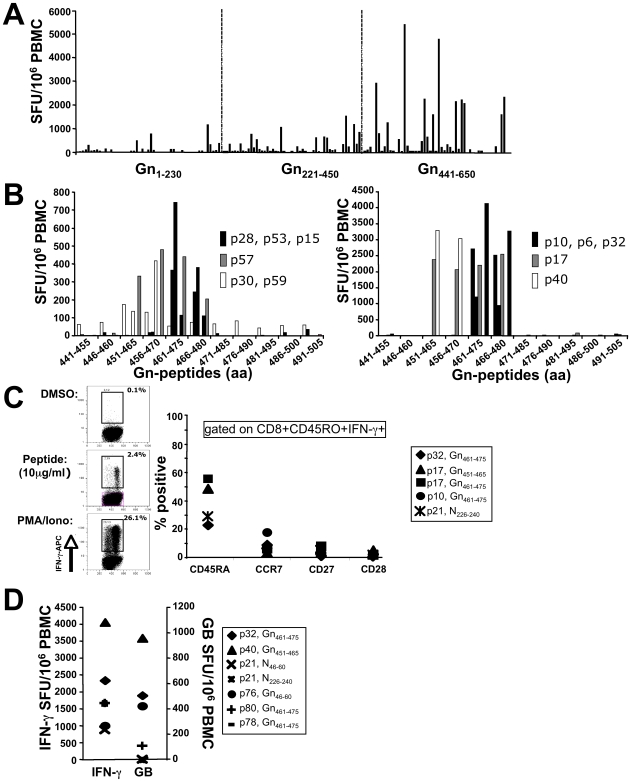
Downmapping of Gn-derived epitopes and characterization of specific T cells. (**A**) Gn-specific T-cell responses among the 51 positive patients, according to amino-terminal (aa 1–230), central (aa 221–450) and carboxy-terminal (aa 441–650) region. Each bar represents the sum of mean triplicate responses towards the peptides representing a given region. (**B**) aa 451–480 of Gn comprises of highly immunogenic epitopes and is recognized by 8/11 patients with strong responses towards the carboxyterminus of Gn. (**C**) Surface marker staining of PBMC of specific, IFN-γ secreting CD8^+^CD45^+^ after stimulation with 10 µg/ml of Gn- and N-derived 15mer peptides, indicating a predominantly end-differentiated phenotype of specific CD8 memory T-cells. DMSO and PMA/Ionomycin were used as control stimuli. (**D**) A high percentage of IFN-γ secreting Gn-specific CD8 memory T-cells readily secretes granzyme B upon *ex-vivo* stimulation with their cognate peptide years after acute ANDV-infection (e.g. p32: 13.2 yrs, p40: 5.4 yrs post hospitalization, respectively).

Subsequently, results from IFN-γ ELISPOT assays revealed that regions Gn_1-230_ and Gn_221–450_ elicited a mean of 102 and 209 SFU/10^6^ PBMC in 34% and 38% of all responsive patients, respectively (**Fig. 3A**). However, Gn_441–650_ elicited a mean response of 623 SFU/10^6^PBMC (range 0–5506 SFU/10^6^PBMC) in 24/51 (46%) patients. These data clearly indicate that epitopes within the carboxyl-terminus of Gn of ANDV are responsible for the immunodominance of Gn among ANDV-convalescent individuals. In contrast to the strong Gn-specific responses, Gc_641–815_ and Gc_806–980_ elicited a response in 24% (77 SFU/10^6^PBMC) and 11% (25 SFU/10^6^PBMC) of all patients, whereas Gc_971–1140_ was targeted by only 4% (30 SFU/10^6^PBMC) of patients (data not shown).

We next stimulated cryopreserved PBMC from patients with Gn_441–650_-specific response using peptide pools representing Gn_441–505_, Gn_496–560_, Gn_551–615_ and Gn_606–650_, respectively. This approach revealed that region Gn_441–505_ comprised the major epitopes of Gn_441–650_ (data not shown). We then challenged cryopreserved PBMC of 11 patients with individual peptides spanning region Gn_441–505_. As shown in Figure 3B, only three patients (p30, p40, p59) exclusively recognized peptides Gn_451–465_ and Gn_456–470_, whereas p17 and p57 additionally recognized Gn_461–475_ and Gn_466–480_. By contrast, six patients (p6, p10, p15, p28, p32, p53) showed exclusive recognition of Gn_461–475_ and Gn_466–480_. Thus, a total of five patients recognized Gn_451–465_/Gn_456–470_, whereas eight patients recognized Gn_461–475_/Gn_466–480_. Subsequently, four additional individuals with exclusive and significant responses towards Gn_461–475_/Gn_466–480_ were identified (data not shown).

Together with a previous report from our lab [23], these epitopes are the first described within the Gn-region of hantaviruses. In addition, we determined immunodominant regions of ANDV N-protein, which included N_1–70_ (24.3% of responsive patients elicited a significant response) and N_121–190_ and N_181–250_ (21.2% and 19.5%, respectively) eliciting mean responses of 113–147 SFU/10^6^PBMC (data not shown). Downmapped individual epitopes within the N-protein are summarized in **Table 1**.

**Table 1:** Determined T-cell epitopes within the ANDV N-protein and observed range(s) of response(s).

Epitope, aa region	Peptide sequence	(Range of) response(s) (SFU/106 PBMC)
N_41–55_	KSTLQSRRAAVSTLE	183
N_46–60_	SRRAAVSTLETKLGE	1760
N_61–75_	LKRQLADLVAAQKLA	120–143
N_106–120_	SIDLEEPSGQTADWK	490–807
N_126–140_	ILGFAIPIILKALYM	116–645
N_131–145_	IPIILKALYMLSTRG	143–464
N_141–155_	LSTRGRQTVKDNKGT	232
N_146–160_	RQTVKDNKGTRIRFK	37–277
N_186–200_	STMKAEEITPGRFRT	83
N_191–205_	EEITPGRFRTIACGL	180–743
N_221–235_	GVIGFGFFVKDWMDR	313
N_226–240_	GFFVKDWMDRIEEFL	1700
N_231–245_	DWMDRIEEFLAAECP	270–360
N_236–250_	IEEFLAAECPFLPKP	150–426
N_241–255_	AAECPFLPKPKVASE	117
N_251–265_	KVASEAFMSTNKMYF	210

We next wondered, which state of differentiation was expressed by the IFN-γ producing memory CD8^+^ T cells. Intracellular cytokine staining showed that IFN-γ^+^CD8^+^CD45RO^+^ T-cells expressed varying levels of CD45RA but consistently expressed a CCR7^−^CD28^−^CD27^−^ effector memory phenotype (**Fig. 3C**). In line with this terminally differentiated phenotype, 25% of all IFN-γ^+^ T cells secreted granzyme B upon stimulation with their cognate peptide (**Fig. 3D**) whereas no IL-2 could be detected (data not shown). In addition, up to 45% of these IFN-γ^+^CD8^+^ memory T cells co-expressed TNF-α as determined by ICS (data not shown). These findings suggest that even years after acute ANDV infection (e.g. p32 and p40 were investigated 5.4 and 13.2 years after hospitalization, respectively), high frequencies of cytolytic memory CD8^+^ T-cells are maintained in the periphery.

### Impact of HLA-B*35- restricted responses on clinical outcome

**Figure 4:**
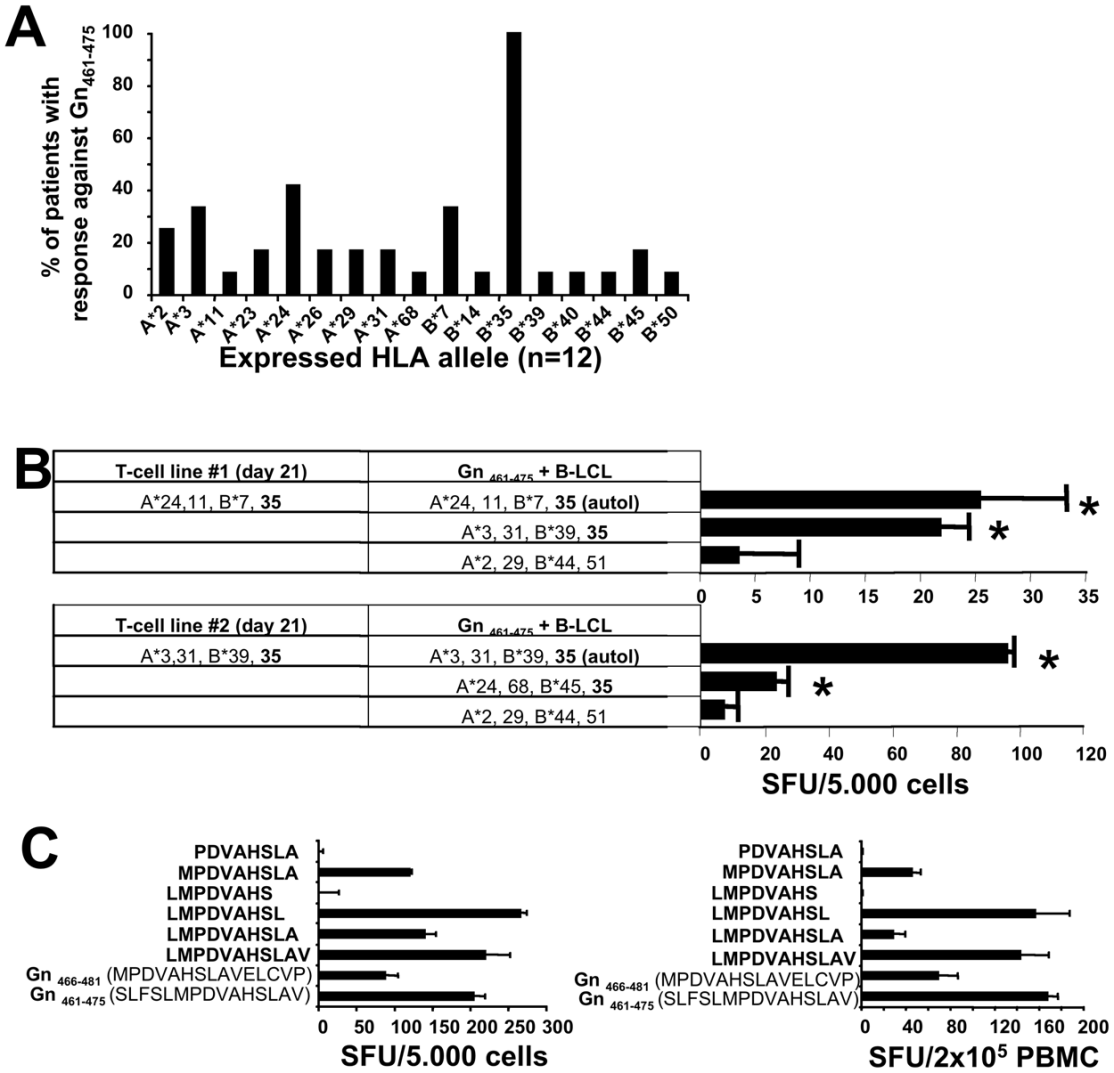
HLA-restriction and minimal optimal epitope of Gn_461–475_. (**A**) Frequency of HLA-A- and B-alleles among individuals with positive response towards Gn_461–475_, indicating that HLA-B*35 is the only allele in common. (**B**) HLA-B*35-restriction of Gn_461–475_. Two Gn_461–475_–specific T-cell lines were generated over 21 days and then stimulated by Gn_461–475_ in the presence of autologous or heterologous APCs (B-LCL). * indicates significant responses (p<0.05). (**C**) downmapping of the minimal epitope of Gn_461–475_ Gn. Truncated versions of Gn_461–475_ were used to stimulate a 21-day Gn_461–475_–specific T-cell line (left panel) derived from a HLA-B*3502-positive donor in an 24 hour IFN-γ ELISPOT assay. Identical results were obtained with cell lines from two HLA-B*3501-positive individuals (data not shown) and in an *ex vivo* ELISPOT assay using PBMC from a HLA-B*3505-positive individual (right panel).

As both the Gn_451–465_/Gn_456–470_ and Gn_461–475_/Gn_466–480_ epitopes share 10 amino acids in sequence within the pairs, we reasoned that one CD8^+^ T-cell epitope may be located within each overlapping sequence, respectively (e.g. Gn_456–465_ and Gn_466–475_). In support of this hypothesis, the analysis of HLA-A, -B, -DR and -DQ alleles revealed that 5/5 patients with response towards Gn_451–465_/Gn_456–470_ exclusively shared the HLA-A*24 allele (data not shown), whereas the HLA-B*35 allele was the only allele shared by all 12 patients recognizing Gn_461–475_/Gn_466–480_ (**Fig. 4A**). These data suggest the existence of two separate but neighboring CD8 T-cell epitopes in the carboxyterminal region of Gn that are restricted by HLA-A*24 and HLA-B*35, respectively. Indeed, as shown in Figure 4B, a significant response in a Gn_461–475_–specific T-cell line could only be detected when the HLA-B*35 allele was present on heterologous APCs (B-LCL).

It was previously suggested that severe HCPS due to SNV is associated with the HLA-B*35 allele [14] and with CD8 T-cell responses restricted to it [13]. We therefore were interested in the relation of memory T-cell responses and outcome of their ANDV infection in HLA-B*35-positive and negative patients (**Fig. S1**). Among all 78 patients, that is patients with (n = 51) and without (n = 27) significant memory T-cell responses, no differences in overall T-cell responses could be observed when comparing HLA-B*35-negative patients with mild or severe HCPS (**Fig. S1A**). By contrast, we found an about 3-fold higher overall T-cell response in HLA-B*35-positive patients with mild HCPS as compared to both HLA-B*35-positive patients with a history of severe disease and either group of HLA-B*35-negative patients (**Fig. S1B**). Likewise, 10/12 (83%) HLA-B*35-positive patients with significant responses to Gn_461-475_ had a history of mild HCPS (**Fig. S1C**). Thus, these data suggest that HLA-B*35-restricted memory T-cell responses are related to mild rather than to severe disease outcome.

We next sought to determine the optimal epitope of Gn_461–475_/Gn_466–480_ (**Fig. 4C**). Because we consistently observed stronger immune responses towards Gn_461–475_ (SLFSLMPDVAHSLAV) than towards Gn_466–480_ (MPDVAHSLAVELCVP), we reasoned that Leucine at position 465 may increase either the binding affinity or the TCR-recognition of the overlapping sequence Gn_466–475_ (MPDVAHSLAV). We therefore decided to generate Gn_461–475_–specific T-cell lines from HLA-B*3501 individuals and then challenged these cells with cleaved peptides of Gn_465–475_ (LMPDVAHSLAV). As shown in Figure 4C, cleavage of the carboxyterminal Leucine at position 473 led to a complete loss of epitope recognition. Similarly, elimination of the aminoterminal Methionine at position 466 was critical for epitope recognition. Most interestingly, virtually identical results were obtained when cells from HLA*B3501, HLA-B*3502 and HLA-B*3505 individuals were challenged with cleaved peptides, indicating that the Gn_466–473_ epitope is equally immunogenic among different HLA-B*35 subtypes.

### High frequencies of memory CD8^+^ T cells that have the potential to exert cytotoxic function are maintained in the periphery

**Figure 5:**
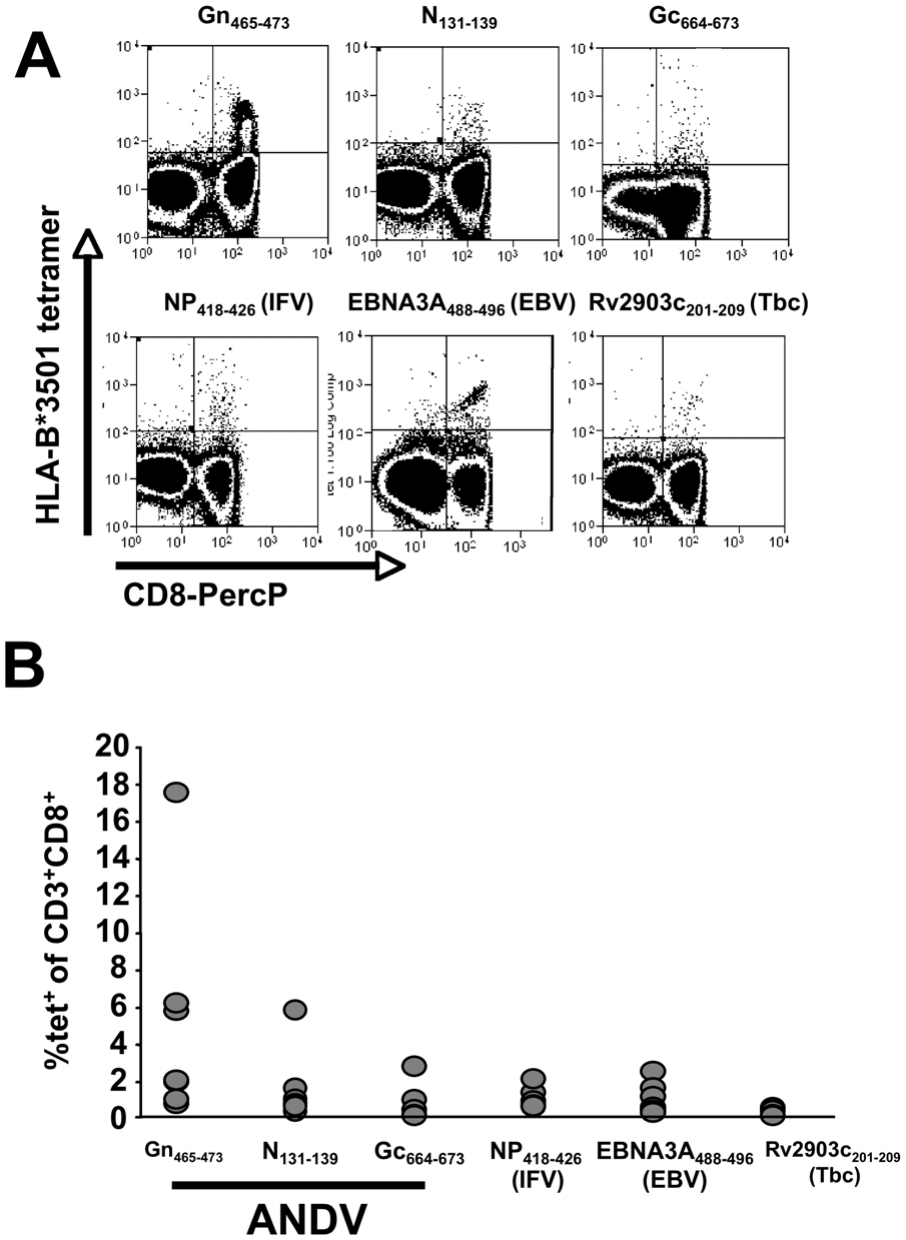
Tetramer analysis of pathogen specific CD8+ memory T cells in seven HLA-B*3501^+^ individuals. (**A**) staining of CD3^+^ cells using HLA-B*3501:tetramer complexes (see table 2). (**B**) Frequencies of tetramer^+^CD3^+^CD8^+^ cells found in seven HLA-B*3501-positive ANDV-convalescent individuals.

We next were interested in comparing the phenotype of Gn_465–473_ restricted T cells and with other HLA-B*3501-restricted virus-specific T cells in seven HLA-B*3501 positive ANDV-convalescent patients (**Fig. 5A, B**). The mean abundance of Gn_465–473_ specific T-cells was higher than those specific for N_131–139_ and Gc_664–673_ –epitopes, described by Kilpatrick et al. [13], the latent EBV-epitope EBNA3A_458–466_ [32], the Influenza A NP_418–426_ epitope [33], or the Rv2903c_201–209_ epitope of *Mycobacterium tuberculosis*, known to be recognized by BCG-vaccinated individuals [34].

In the seven HLA-B*3501-positive individuals we had detected between 0 and 4394 SFU/10^6^ PBMC (0%–0.0044%) following stimulation with peptide Gn_461–475_ in IFN-γ ELISPOT assays. When normalizing the results by the percentages of CD3^+^CD8^+^ cells in these individuals (range 9.9–33.9% of PBMC, mean 19.7%), one would have expected between 0% (p45) and 0.036% (p17) of CD3^+^CD8^+^ cells being tetramer positive. However, we found 0.3% and 5.9% of all CD3^+^CD8^+^ T cells being positive for Gn_465–473_:HLA-B*3501 tetramer complexes, respectively. In addition, the highest frequencies for tetramer-positive cells were found in patient 10 (16.8% of CD3^+^CD8^+^ cells), whereas only 0.0111% of his CD3^+^CD8^+^ cells produced IFN-γ in ELISPOT assays. This discrepancy between both detection methods is in line with previous reports [35].

**Figure 6:**
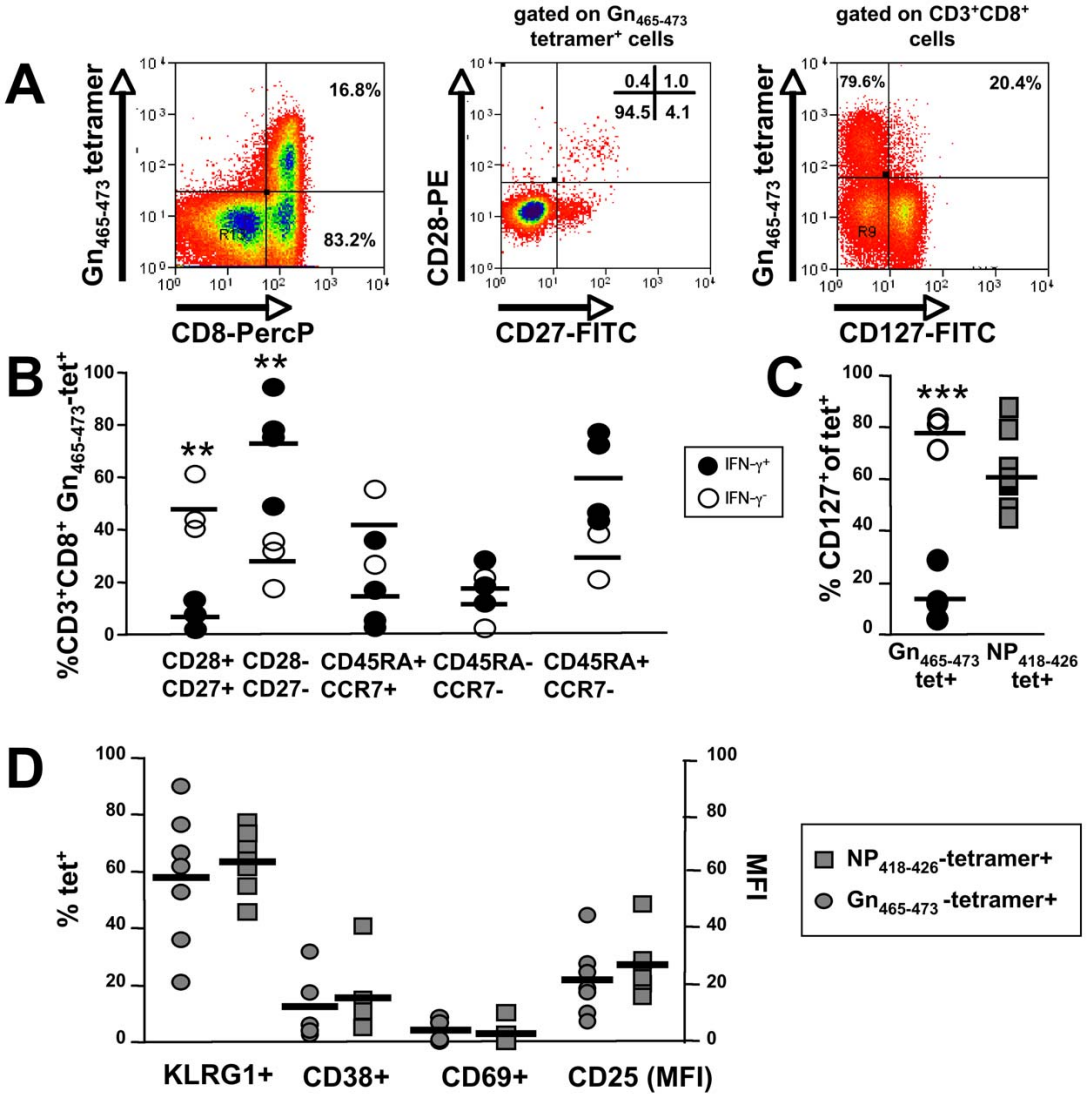
Phenotypic analysis of Gn_465–473_-specific subsets and comparison with (Influenza) NP_418–426_-specific CD8^+^ T cells. (**A**) Gn_465–473_:tetramer^+^CD3^+^CD8^+^ cells predominately express a a CD28^−^CD27^−^CD127^−^ phenotype. (**B**) Patients with Gn_461–475_-specific responses in IFN-γ ELISPOT (n = 4) assays show significantly more (p<0.01) highly differentiated CD28^−^CD27^−^ Gn_465–473_:tetramer^+^CD3^+^CD8^+^ cells than those individuals without IFN-γ responses (n = 3). No significant differences were observed as regards to expression of CD45RA and CCR7. (**C**) Patients with Gn_461–475_-specific responses in IFN-γ ELISPOT (n = 4) assays show significantly less (p<0.001) CD127^+^ Gn_465–473_:tetramer^+^CD3^+^CD8^+^ cells than those individuals without IFN-γ responses (n = 3). By contrast, no clear differences were observed as regards to Influenza-specific T-cells of the same individuals. (**D**) No signs of significant activation could be observed in Gn_465–473_:tetramer^+^CD3^+^CD8^+^-specific cells as when compared to Influenza A NP_418–426_:tetramer^+^CD3^+^CD8^+^-specific cells.

We next determined the state of differentiation of Gn_465–473_-specific T-cells, where a clear dichotomy was observed (**Fig. 6A, B**). In patients with positive responses towards Gn_461–475_ in IFN-γ ELISPOT assays (IFN-γ ^++^), Gn_465–473_ specific T cells were mostly CD45RA^+^CCR7^−^ and significantly more of a differentiated CD28^−^CD27^−^ phenotype as compared to IFN-γ^−^ samples. In addition, we found significant differences with regard to the IL-7Rα (CD127), which is crucially involved in maintenance of memory T-cells in the periphery in the absence of cognate antigen [36]. Patients with IFN-γ^+^ ELISPOT results showed mainly CD127^−^ Gn_465−473_ T cells, whereas T cells of IFN-γ^−^ patients were mostly CD127^+^ (**Fig. 6A, C**). Thus, Gn_465−473_-specific CD8^+^ T cells showed a phenotype that is clearly distinct relative to that described for other self-limited diseases such as those caused by influenza A and respiratory syncytial virus but more resembled the pattern associated with latent infections, such as past exposure to CMV [37]. Because a CD28^−^CD27^−^CD127^−^ phenotype was previously described to be a result of ongoing antigen-stimulation, as found in latent CMV infection, we next determined the expression of activation markers, such as of KLRG-1, CD69, CD38 and CD25 on Gn_465–473_ and Influenza A-specific T cells within the seven HLA-B*3501^+^ ANDV-convalescent (**Fig. 6D**). No significant differences could be observed between IFN-γ^+^ and IFN-γ^−^ Gn_465–473_-specific populations or between Gn_465–473_- and Influenza A NP_418–426_-specific T-cells.

### Prospectively high frequencies of Gn-specific memory T cells, anti-N antibodies and neutralizing antibodies

**Figure 7:**
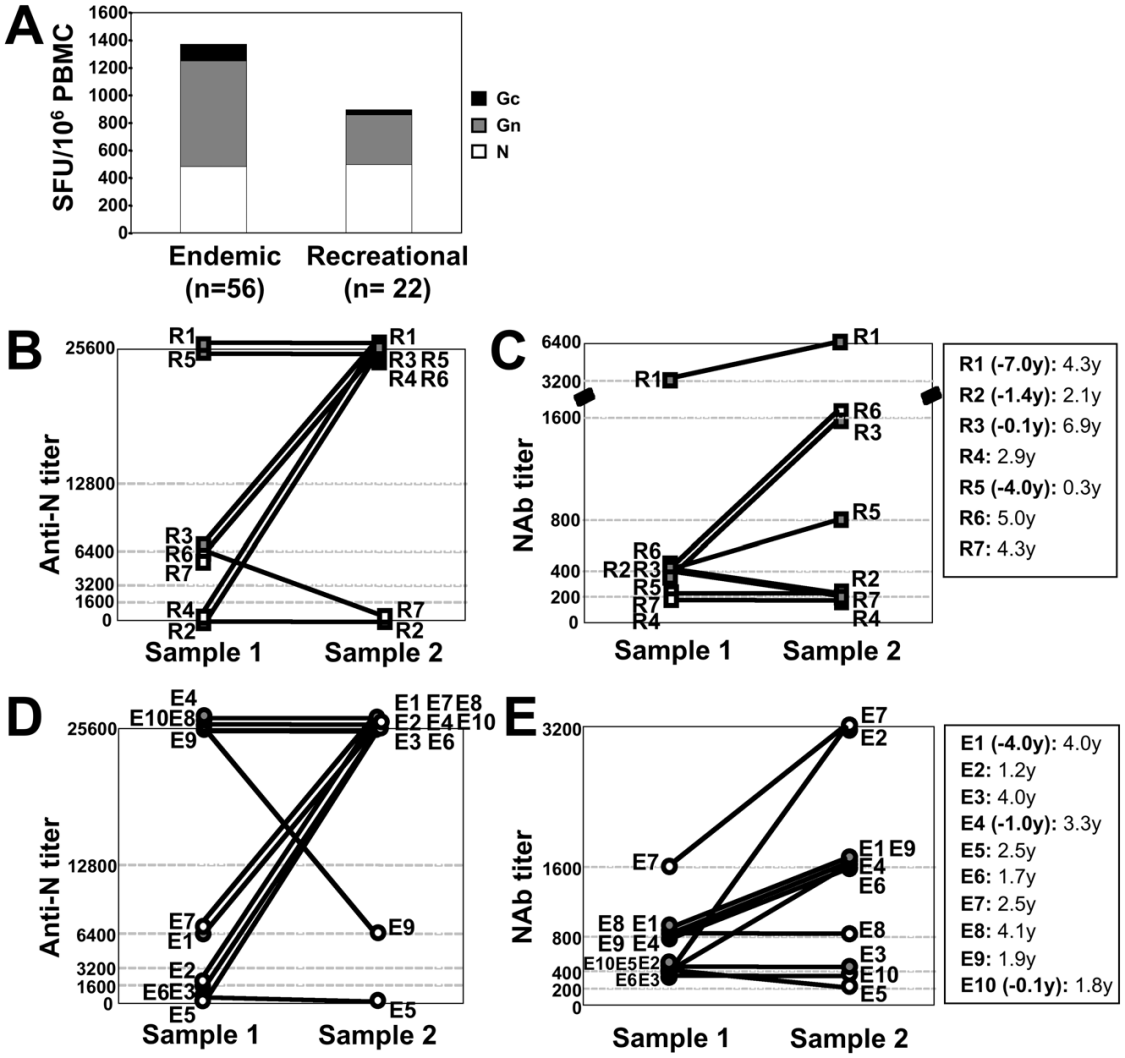
Comparison of immune responses between patients residing in endemic areas (E-patients) and those who got infected during recreation (R-patients). (**A**) no significant differences in memory T-cell responses between E- and R-patients. (**B-E**) prospective anti-N (determined by indirect ELISA) and NAb titers (determined by FRNT) in R-patients (**B, C**) and E-patients (**D, E**), respectively. Symbols filled in grey indicate patients in who sample 1 was taken months or years after the acute infection; the time difference between the acute phase and the timepoint of sample 1 is indicated as negative number in parenthesis (legend on the right). The second number indicates the time period between sample 1 and sample 2, respectively.

In a next step we assessed whether re-exposure to viral antigens could have led to a boost in the donor's immune response. We therefore compared memory T-cell responses in patients who got infected during recreation (R-patients) with those of residents in endemic areas (E-patients) (**Fig. 7A**). No significant differences were observed between the two groups in those responses, although endemic patients revealed about double as many Gn-specific memory T-cells than recreational patients (mean 765 vs 361 SFU/10^6^ PBMC) for unclear reasons. However, these results are not in line with the hypothesis of repeated viral exposure in patients who reside in endemic areas, since N- and Gc-specific responses were virtually identical in both groups.

In addition, we identified seven individuals, which had been infected during recreation (R-patients, **Fig. 7B, C**) and ten individuals residing in endemic areas (E-patients, **Fig. 7D, E**), for all of which two prospective serum samples were available. The time period between sample 1 and sample 2 was 0.3–6.9 years and 1.2–4.1 years in R- and E-patients, respectively. Surprisingly, in R- and E-patients anti-N titers raised four- to 64-fold between samples 1 and 2 in 4/7 and 5/10 patients, respectively. Most intriguingly, however, also neutralizing antibody (NAb) titers rose two- to eight-fold in 4/7 and 6/10 of R- and E-patients, respectively. Importantly, NAb titers, measured by a blinded worker, increased two- to four-fold in patients R1, R3, R5, E1 and E4 between sample timepoint 1 and 2, although in all cases sample 1 was taken months to years after the acute phase. Taken together, these results suggest that re-exposure to extrinsic, environmental virus is not responsible for the observed rise in NAb titers or high frequencies of memory T cells.

**Figure 8:**
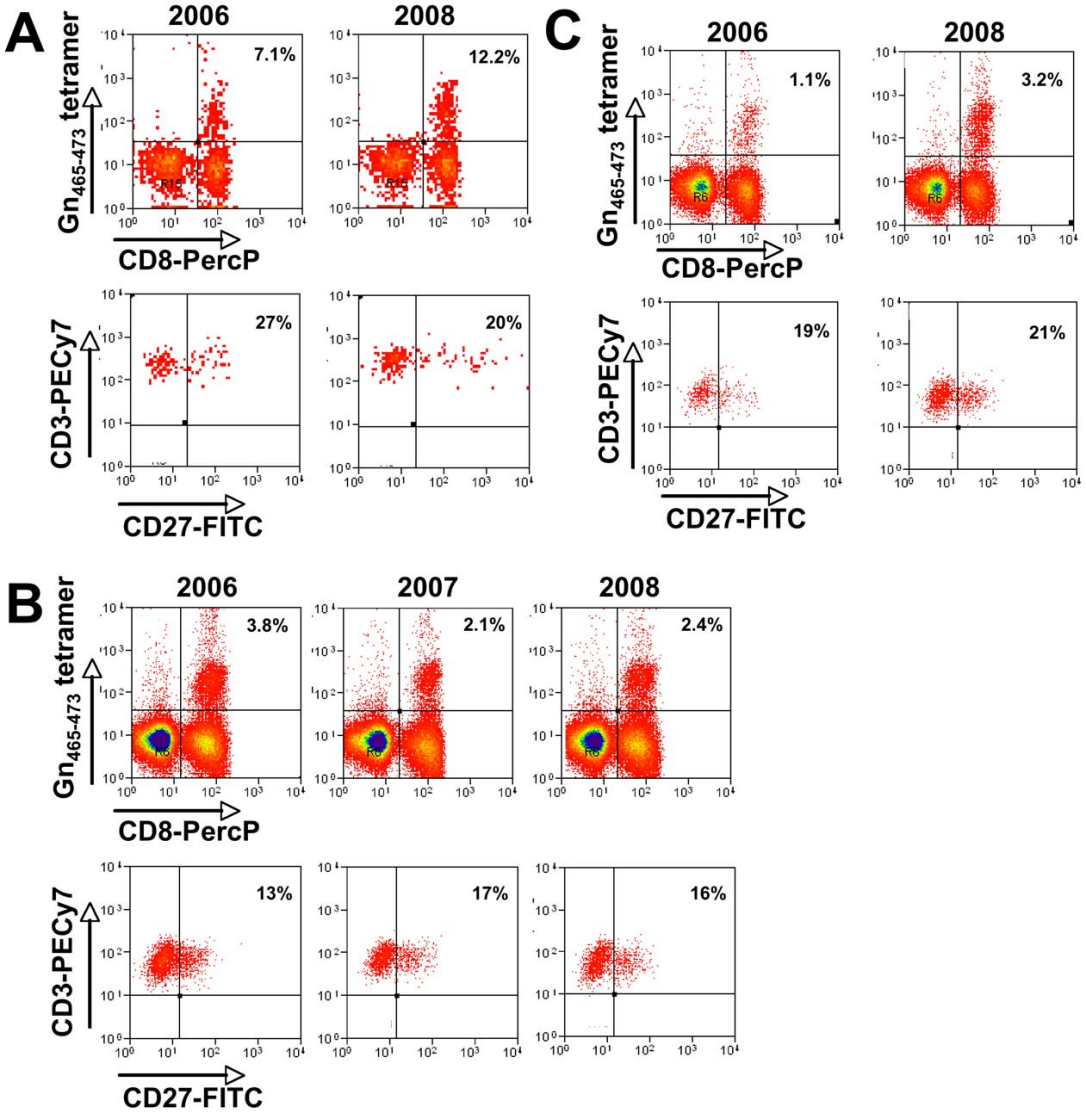
Prospective analysis of Gn_465–473_–specific T cells in three HLA-B*3501^+^ individuals. Cryopreserved PBMC samples of each donor (**A–C**) and year were thawed and processed in parallel and assayed together in order to guarantee optimal comparability of the samples. Upper panels of each patient are gated on PI^−^CD3+ T-cells, lower panels show CD27 expression gated on tetramer^+^CD3^+^CD8^+^ cells, respectively. Note that (**A**) is showing frequencies of patient E9 and (**C**) of patient E2 (compare with Figure 7E).

Finally, we sought to prospectively study Gn-specific T-cells in three patients. When Gn_465–473_-specific T cells were phenotyped over a time period of two years (**Fig. 8A–C**), no dynamic changes of the CD27^−^ population could be observed, indicating that differentiated Gn_465–473_-specific T cells are able to stably persist at high frequencies without the need for B7:CD28- or CD70:CD27-mediated survival signals. However, in patients E9 and E2 (**Fig. 8A, C**, respectively), Gn_465–473_-specific T cells actually increased over time, paralleling the two- to eight-fold increase in NAb titers observed in these two individuals (**Fig. 7E**).

## Discussion

Infection with ANDV is the predominant cause for HCPS in South America. Case-fatality rates of currently 36%, person-to-person transmission and the absence of a proven effective antiviral treatment urge the development of a vaccine. Although the protective potential of neutralizing antibodies against the hantavirus surface glycoproteins Gn and Gc, but not the N-protein, was established *in vitro* [38]–[40] and in animal models [41]–[43], efforts to induce long-lasting neutralizing antibodies in human volunteers have been unsuccessful so far [29],[44] or remain to be proven effective and long lasting [45].

On the other hand, several early reports highlight the importance of lymphocytes for immunity of mice towards hantaviruses, such as HTNV [15]–[17]. Likewise, appearance of virus-specific CD8^+^ T cells with cytotoxic activity and the ability to produce IFN-γ and TNF-α was associated with clearance of HTNV in newborn mice. In contrast, HTNV infection was not cleared when TNF-α production and cytotoxic activity of specific CD8^+^ T cells were impaired [18]. In another report, (HTNV-) N-protein-specific CD8^+^ memory T-cells, induced by a DNA vaccine, conferred partial protection against re-challenge with a vaccinia virus expressing the N-protein [19]. One study in mice showed that N-protein specific T cells rather than antibodies mediated protection and cross-protection upon re-challenge with homologous and heterologous hantaviruses [20]. Finally, ANDV infection of Syrian hamsters - the sole animal model for human HCPS–could be prevented for at least 10 months by previous vaccination with ANDV N-protein [21], again indicating that protection can be achieved independently of neutralizing, Gn/Gc-specific antibodies. Most recently Safronetz et al. confirmed these findings in Syrian hamsters vaccinated with Gn-protein expressing Adenovirus vectors. Interestingly, these animals were protected from lethal ANDV infection independently of neutralizing antibodies and showed no or very low levels of ANDV-RNA up to 9 days after ANDV infection [22]. As for ANDV-infection in man, we recently showed that clearance of ANDV-RNA from peripheral blood cells was closely related to the appearance of cytotoxic CD8^+^ T cells, but not NAb, in a patient about two months after the acute infection [23].

Taken together, these reports suggest that cytotoxic T cells are crucially involved in clearance and protection from hantaviruses. Conversely, establishment of hantavirus-specific cytotoxic memory CD8^+^ T cells prior to infection, e.g. by a vaccine, may provide protective, albeit not sterilizing, immunity to the host. However, limited information on human cellular immunity to hantaviruses is available and, to date, only one study addresses ANDV-specific T-cell responses [23].

Using a panel of 310 overlapping peptides spanning the entire N-, Gn- and Gc-protein of ANDV allowed us to study most, existing T-cell epitopes in 78 convalescent survivors of ANDV infection in a non-HLA-restricted manner. In contrast to other reports with a similar approach, the majority of responses were specific for Gn- but not N-protein-derived epitopes. Thus, our results in ANDV-infected patients seem to contradict the current dogma of N-protein being the principal T-cell immunogenic hantavirus antigen [19], [20], [22], [24], [25], [27], [46]-[48]. However, in previous studies, epitope-specific T cells were detected either by *in vitro* expansion prior to testing or using individual peptides or tetramer complexes for *ex vivo* detection in a small and HLA-selected patient populations [13], [24]–[28]. Thus, differences in the experimental design, rather than its elevated immunogenicity, may explain, why we found Gn being the immunodominant antigen of ANDV, whereas no single Gn-epitope had been described for other hantaviruses. In fact, 92–96% of the amnio acid sequence of the two Gn-epitopes described herein, are conserved within the PUUV and SNV sequence, respectively. An alternative explanation may derive from the differences between our study and other studies in the timing of T-cell testing after the acute phase or differences in infection kinetics between the different hantaviruses. Specifically, as can be seen in Figure 2D, N-derived epitopes seemed to be relatively predominant up to four years after the acute infection, whereas Gn-derived epitopes were predominant in patients with a longer convalescence phase. In addition, the kinetics of NAb titers in our patients suggest that viral antigen may be present for months or years after the acute infection, a phenomenon which has not been described for other hantaviruses. Thus, differences in epitope avidity and/or precursor expansion over time may have contributed to the relative predominance of Gn-specific T-cells in our study.

Of note, 80% of all patients with detectable T-cell responses recognized Gn-derived epitopes (Fig. 1B). To our surprise, however, responses against Gn were not broad but rather focused to the carboxyterminus of Gn, namely the region of aa 451–480. Interestingly, the cytoplasmic tail of Gn has been shown to contain important virulence factors as it suppresses type 1-interferon responses in infected cells [49],[50]. On the other hand, the carboxyterminal 142 residues of pathogenic (namely of ANDV and HTNV), but not non-pathogenic hantaviruses, prone the C-terminal tail of Gn towards degradation by the proteasome, which then leads to the presentation of epitopes by MHC I molecules to CD8^+^ T-cells [51]. This mechanism could explain the relative immunodominance of Gn-derived epitopes seen in our study and also may represent a virulence factor of ANDV suggesting that T cells are causative for HCPS. Nonetheless, an early and vigorous cytotoxic T-cell response towards epitopes of the C-terminal Gn may also be able to restrict the virulence of ANDV infection. Indeed, among HLA-B*35-positive patients mild disease outcome seemed to be associated with stronger responses towards the Gn-carboxyterminus than in patients with severe HCPS (Fig. S1B, C). In line with this finding, a recent study in 87 Chilean ANDV-infected patients found that the HLA-B*35 allele was the most frequent allele among patients with mild disease and almost twice as frequent as in patients with severe disease [52]. Although these data seem to contradict previous reports describing both the expression of the HLA-B*35-allele [14] as well as HLA-B*35-restricted T-cell responses [13] as risk factors for severe HCPS by SNV, it remains speculative whether our results indicate an pivotal role for T cells in disease outcome. The size of the memory T-cell pool is only indirectly linked to the effector T-cell response by the original burst size [53] and therefore may not reflect the size and composition of the effector T-cell pool during the acute phase. Moreover, 33% of HLA-B*35-negative patients and 48% of patients without any detectable memory T-cell responses had a history of mild HCPS (data not shown). Likewise, 52% of patients with severe HCPS did not show memory T-cell responses (data not shown). Both seem to argue against an exclusive role of T cells for disease outcome. In addition, other hand, we also showed a discrepancy between ELISPOT and tetramer-derived T-cell frequencies (see above), which indicates the existence of IFN-γ-negative ANDV-specific T-cells. In fact, three of the seven studied HLA-B*3501 positive donors did not show significant responses in initial IFN-γ ELISPOT assays (Fig. 1) but showed substantial numbers of tetramer-positive CD3^+^CD8^+^ T cells. This is in line with previous reports comparing determination of T-cell frequencies by ELISPOT and tetramer analysis [35]. In this report tetramer analysis revealed on average ten-fold higher frequencies than IFN-γ ELISPOT assays of T cells specific for a HLA-A2-restricted HIV Gag-derived peptide. This report as well as our data suggests that the vast majority of virus-specific T cells may not readily secrete IFN-γ when stimulated by peptides in ELISPOT assays. It is also possible that these T cells were functional during the acute phase but not during convalescence. Alternatively, differences in the infectious dose (e.g. low versus high infectious dose) or the kinetics and doses of the evolving neutralizing antibodies may interfere with the functional quality of the memory T-cell pool. Taken together, additional studies with HLA-tetramers in acutely ANDV-infected patients will be necessary to better understand the role of HLA-B*35 and T-cell kinetics for ANDV disease outcome.

Another interesting aspect of our study concerns the longevity of the memory T cells in association with their highly differentiated phenotype. After more than 13 years after infection we still detected 564–2152 SFU/10^6^ PBMC by ELISPOT, most of which were directed towards Gn-derived epitopes.

With regards to the longevity of CD8 memory T cells, our results nicely confirm a previous study by Van Epps et al. in PUUV-convalescent patients, in which up to 100–300 SFU/10^6^ PBMC of N-epitope-specific CD8 T cells were found in three patients up to 15 years after acute infection [24],[25],[27]. However, by tetramer analyses we detected still-higher frequencies with up to 16.8% of all CD3^+^CD8^+^ T cells proving positive for the single epitope Gn_465–473_ while displaying a late effector memory phenotype (CD127^−^CD28^−^CD27^−^CCR7^−^). This phenotype was in line with the cells' ability to readily secrete granzyme B and TNF-α without IL-2. Surprisingly, we also found that up to 5% of tetramer^+^ cells, which, again in accordance with their CD28^+^CD27^+^CCR7^+^CD127^+^ phenotype, did not exert any immediate effector functions, such as IFN-γ secretion upon stimulation with their cognate epitope. While these data again show that screening by ELISPOT underestimates the real proportion of Gn-specific T cells [35], it is also tempting to speculate, albeit virtually impossible to prove, that patients with either of the observed phenotypes differ at their stage of immunity towards a possible ANDV re-challenge.

In absence of antigenic stimulation as well as of autocrine or paracrine IL-2 and co-stimulation via CD28:B7 and CD27:CD70 interaction, late effector memory T-cells are heavily prone to apoptosis. However, we herein were able to show in three HLA-B*3501^+^ patients that peripheral Gn_465–473_-specifc T cells were maintained in the periphery for at least two years, despite a consistent CD27^−^ phenotype, thereby lacking the receptor for crucial anti-apoptotic signals provided by CD70. While the former perception was that senescent end-differentiated CD28^−^CD27^−^ T cells were unable to divide, recent evidence suggests, that highly differentiated granzymeB^+^CD8^+^ memory T cells are actually dividing upon stimulation equally well as naïve CD8^+^ T cells [54]. In man, the CD45RA^+^CD28^−^CD27^−^CCR7^−^ late effector memory phenotype has been mainly described in patients with latent CMV infection [37],[55]. In this model repetitive or latent antigen stimulation is supposed to drive CD8^+^ memory differentiation and/or the recruitment of new memory T-cells. However human hantavirus infections are not known to cause latent or chronic infections and we were not able to detect viral RNA in plasma or peripheral blood cells of patients with sustained and high T-cell responses (data not shown). Also, we failed to detect a fingerprint of recent antigenic stimulation of tetramer-positive cells through assessment of the expression of additional activation markers, including CD69, CD38 and CD25. In addition, expression of IL-7Rα (CD127) was described to be a critical factor for long-term survival of CD8^+^ memory T cells in absence of their cognate antigen. Since CD127 is usually downregulated upon antigen exposure and rapidly re-expressed after antigen clearance, it is consistent that mainly virus-specific CD127^+^CD8^+^ memory T-cells are found in studies on Influenza-, respiratory syncytial virus- and HBV- specific T-cells. By contrast, in persistently HIV-, CMV- or EBV-infected individuals T cells are maintained despite their lack of CD127 expression [56],[57]. Finally, KLRG1 is mainly expressed on antigen-experienced T-cells with immediate effector functions [55]. Thus, considering ANDV a self-limiting transient infection in man, a CD127^+^KLRG1^−^ phenotype would have been expected years after the infection. However, IFN-γ ^+^, but not IFN-γ ^−^, Gn_465–473_-specific T cells expressed substantially less CD127 than their Influenza A virus (NP_418–426_)-specific counterparts, whereas no clear pattern could be observed regarding the KLRG1 expression. However, although no difference could be observed between ANDV (Gn_465–473_) and Influenza (NP_418–426_)–specific T-cells with regards to CD25, CD38 and CD69 expression, the lack of CD127 suggests persistent antigenic stimulation in individuals with IFN-γ^+^ T cells. When employing BLAST, we were not able to identify other organisms that share the amino acid sequence of Gn_465–473_, thereby making cross-reactivity an unlikely explanation. Consistently, no further serology testing was performed in convalescent patients.

We next hypothesized that residents of endemic areas might have received intermittent antigen-boosters due to viral re-exposure and therefore should show somewhat higher T-cell responses to all viral antigens. However, we did not find significant differences in ANDV-specific T cell numbers when comparing patients who reside in endemic areas and those, which got during recreation got infected in an endemic region (Fig. 7A), while residing in non-endemic areas.

Moreover, in the majority of prospective serum samples of ten patients from endemic regions and of seven patients from non-endemic regions, we surprisingly found an increase in both anti-N and NAb titers despite the fact that the second sample was taken years after the first samples in most cases. The fact that this was also observed in patients who never had returned to endemic regions since their primary ANDV infection, suggests that re-exposure to extrinsic (environmental) virus does not account for high antibody titers and, conversely, not for high ANDV-specific T-cell frequencies. Regarding the increase in NAb titers between sample 1 and 2, we cannot exclude that NAb titers continued to rise after sample 1 was drawn during or shortly after the acute phase. Therefore, it is possible that in these patients (Fig. 7) titers of sample 2 in fact were identical or even lower than the maximum titer achieved during the acute phase. However, NAb titers of 13/17 individuals were still relatively high (≥1∶400) at the timepoint of sample 2, that is 1.2–11.3 years after the acute infection. This argues for continuous antigen-exposure in both E- and R-patients, since NAb titers, in contrast to non-neutralizing antibodies, strictly depend on the presence of their cognate antigen. Specifically, in absence of antigen, murine NAb titers fall below the detection limit after 100–200 days [58]. Most importantly, however, we also found that NAb titers increased two- to four-fold in five patients (R1, R3, R5, E1 and E4, Fig. 7C, E) in which sample 1 was taken months to years after the acute phase. Since not only maintenance [58] but also kinetics of NAb titers heavily depend on the presence of viral surface antigens [59], these results support the hypothesis that re-exposure to viral surface (Gn/Gc) antigen is responsible for high and rising NAb titers in both R- and E-patients. Due to the fundamental differences between R- and E-patients in their risk for re-exposure to extrinsic virus, this is turn suggests that intrinsic viral antigen is responsible for the relative “immune-inflation” and the terminal differentiation of Gn-specific CD8^+^ T-cells. Thus, it may be that intermittent release of low doses of viral antigen from intrinsic virus (e.g. that never completely cleared from tissue reservoir(s)) is sufficient to maintain and boost of NAb titers and T-cell frequencies, whereas changes in activation marker expression on ANDV-specific T-cells are too short-lived (with the exception of low CD127 expression) to be consistently different (e.g. KLRG1) from that of Influenza A virus-specific T cells. However, as long as viral antigen or genome cannot be detected in convalescent patients as those described herein, the concept of latent or persistent ANDV infection in convalescent patients remains speculative. Future studies should therefore focus on antigen detection in tissues (e.g. surgical or post-mortem specimen) from solid (e.g. lung, kidney) and immuno-privileged (e.g. brain) organs.

Our data suggest that long-lived effector memory T cells can be maintained at high numbers in the periphery over years independently of IL-7. Notably, our findings resemble those found in murine Sendai and Influenza A virus infections, where epitope-specific T-cell clonal expansions occurred in absence of antigen throughout the CD8 memory pool [60]. As in our study, but in contrast to models of persistent infection, clonally expanded effector memory T-cells in these studies retained potent functionality despite their highly differentiated phenotype. Although we could not formally show clonal expansion for all our patients (with the exception of two individuals, see Fig. 8A, C) due to our non-prospective study design, similar underlying, but yet undefined, mechanisms may explain our findings in human ANDV infection.

The induction of a highly differentiated, resting e.g. Gn_465–473_–specific, memory T-cell subset might be of major interest in the context of vaccine development for several reasons. First, years after infection high numbers of these CCR7^−^ Gn_465–473_–specific cells remain available for immuno-vigilance in the periphery. Second, as shown, this subset possesses the ability to readily secrete antiviral (e.g. IFN-γ, TNF-α) as well as lytic (granzyme B) effector molecules. Third, although most human studies seem to focus on epitopes restricted to HLA-A*02 because of its wide distribution among the Caucasian population (about 25%, [61]), it should be noted that within the Amerindian population, the frequency of HLA-B*35 positive individuals is about 70% higher than in Caucasians [61]. In fact, 25% (range 22–30%) of the inhabitants in ANDV endemic regions in Southern Chile express the HLA-B*35 and/or the HLA-A*02 allele, respectively [52]. Thus, for this population, HLA-B*35-restricted epitopes, like Gn_465–473_, might be of similar impact as HLA-A*02-restricted epitopes. However, it first has to be established in future studies (e.g. Syrian hamster models) whether and to which extend Gn-derived T-cell epitopes may contribute to protective immunity. Furthermore, although hantaviruses are not known to mutate, additional epitopes have to be identified in future studies in order to prevent failure of a T-cell based vaccine due to mutations within the Gn_465–473_ epitope.

Taken together, our results suggest that infection with ANDV may lead to a strong highly differentiated effector memory response. The findings concerning the predominant immunogenicity of ANDV-Gn protein may have implications for the understanding of immunity not only to ANDV, but also to other hantaviruses.

## Materials and Methods

### Patients, clinical classification and samples

A total of 78 patients were enrolled between 4 months and 13.2 years after hospitalization due to either mild or moderate/severe HCPS. All patients had a previous confirmed hantavirus diagnosis done in Chilean reference laboratories by IgG serology to SNV and ANDV antigens by enzyme-linked immunosorbent assay (ELISA), as previously described [62]–[64].

Mild HCPS was defined by the sole support of the patient by symptomatic therapy, including respiratory support by an oxygen mask. On the other hand, ANDV-infected patients who required intensive care by mechanic ventilation and/or anti-shock treatment with vasoactive drugs were defined as moderate/severe HCPS.

All patients included were Chilean citizens and volunteered to participate without receiving monetary incentive. Prior to enrollment all patients enrolled signed informed consent, which was previously informed by IRB committees of Clínica Alemana de Santiago, the Chilean Ministry of Health and regional IRB committees. Before enrollment, patients were extensively informed about the intention of the study by the local study nurse. Upon enrollment patients did not suffer from any signs of active disease and were only enrolled if considered healthy donors. Samples consisted in 45 cc of peripheral venous blood, using tubes containing Sodium Heparin (BD vacutainer). Samples were shipped within 24 hours to our laboratory and were processed immediately upon receipt. PBMC were isolated by Ficoll-Hypague gradient and fresh PBMC were applied to ELISPOT assays. PBMC, which were not used immediately were cryopreserved in liquid nitrogen.

### ELISPOT assays

96-well filterplates (Millipore) were coated with 5 µg/ml of anti-hIFN-γ (Endogen, clone M700A) or 15 µg/ml anti-granzyme B (mabtech, clone GB10) at 4°C overnight one day prior to the assay. For granzyme B assays, prior to coating membranes were activated by incubation of the wells with 15 µl/well of 35% Ethanol for 1 minute. After washing and blocking of the plate, fresh or cryopreserved PBMC or T-cell lines were applied and incubated for 20 hours in an incubator (Nuraire) at 37°C and 5% CO_2_ in the presence of 310 overlapping 15mer peptides (Mimotopes, Australia) organized in 13 pools of 12 to 44 peptides (final concentration 1 µg/ml, each) of continuous sequence spanning the entire genome of the N and GPC protein of the Chilean ANDV [30]. For mapping experiments, cells were incubated with 10 µg/ml of each individual peptide. As negative controls, corresponding dilutions of DMSO (Sigma) were used, whereas a 1∶100 dilution of PHA (M form, Invitrogen) was used as positive control. After the incubation period, plates were washed and incubated with biotinylated secondary antibodies (IFN-γ: clone M701B, granzyme B: GB11) according to the manufacturer's manual. After incubation with Streptavidine-Alkaline phospahtase (Vector, at 1∶1000 for 2 hours), plates were incubated with NCIP/BPT substrate (BioRad), and analyzed using the ELI.Scan (A.EL.VIS GmbH) analyzing unit. Results were expressed as Spot Forming Units (SFU), representing the numeric difference between specific spots and the spots in the negative control (DMSO).

### Focus Reduction Neutralization Test (FRNT)

All FRNT studies were carried out in an approved (C20041018-0267) biosafety level 3 laboratory. Plasma samples from the patient were serially diluted in fourfold increments, mixed with equal volumes of approximately 60 focus forming units (f.f.u.) of a human Chilean virus isolate [65] before incubation on Vero E6 cells, processed and analyzed as described before [66]. The neutralization activity of an antibody was expressed as the highest plasma dilution capable of reducing the number of foci by at least 80%.

### Intracellular cytokine staining

Cryopreserved PBMC of four different patients were challenged *in vitro* for 1.5 hours by a previously determined individual immunogenic peptide (10 µg/ml), the corresponding DMSO dilution or PMA/ionomycin (500/50 ng/ml) in the presence of 1 µg/ml anti-CD49d (clone 9F10) and anti-CD28 (clone CD28.2) (both BD Pharmingen), respectively, and cultured for 4.5 additional hours in the presence of GolgiStop/monensin. Finally, intracellular cytokine staining was performed by fixation, permeabilization of cells and subsequent staining for surface markers and intracellular IFN-γ and TNF-α according to the manufacturer's protocol (BD, California).

### Gn_461–475_-specific T-cell lines

2×10^5^ PBMC/well were stimulated with 10 µg/ml of the Gn_461–475_ in the presence of 10 ng/ml IL-7 and 300 pg/ml IL-12 (R&D systems). On day 2 after setup and every 3–4 days 10 U/ml and 150 µg/ml IL-15 were added to the culture. On day 7 and 14, T-cells were re-stimulated using irradiated (30Gy) PBMC or irradiated autologous (100Gy) B-LCL. On day 21 T-cells were assayed in IFN-γ ELISPOT assays using truncated peptides as indicated.

### Flow cytometry, antibodies and tetramers

Was performed on a 4-colour FACSCalibur (Becton Dickinson) or CyAn (Dako) and using either CellQuest (Becton Dickinson®) or Summit 4.0 (Dako) analyzing software. We used the following antibodies (all BD Pharmingen): CD3-FITC (UCHT1), CD4-FITC (SK3), CD45RA-FITC (HI100), CD27-FITC (M-T271), TNF-α-FITC (Mab11), CD45RO-PE (UCHL1), CD28-PE (L293), CD8-PercP (SK1), IFN-γ-APC (B27), CD3-APC (UCHT1). FITC-, PE- and APC- (MOPC-21) as well as PercP- (X40) conjugated mouse IgG1κ were used as isotype controls. Anti-KLRG-1-Alexa488 was kindly provided by Prof H.P. Pircher (University of Freiburg, Germany). Tetramer complexes were custom-synthesized by the NIAID tetramer facility (Gaithersburg, MD) according to the published protocol (http://research.yerkes.emory.edu/tetramer_core/protocol.html), and Gn_465–473_:tetramers were either APC- or PE-labeled. All other tetramer complexes were APC-labeled. **Table 2** shows a summary of HLA-B*3501 tetramer complexes used in the present study.

**Table 2:** HLA-B*3501 tetramer complexes used in the study.

MHC I tetramer	Peptide sequence	Reference
ANDV Gn465–473	LMPDVAHSL	-
ANDV Gc664–673	TAHGVGEIPM	[13] a
ANDV N131–139	IPIILKALY	[13] a
IFV NP418–426/1980	LPFEKSTVM	[33]
EBV EBNA3A458–466	YPLHEQHGM	[32]
Tbc Rv2903c201–209	EPYLDPATM	[34]

a original epitope sequence derived from Sin Nombre Virus.

### HLA typing

The patients were genotyped for the HLA loci A, B, DRB1 and DQB1, using the SSP PCR (Sequence Specific Primer–Polymerase Chain Reaction) technique. Low and high resolution SSP kits from Dynal (Oslo, Norway) and Invitrogen Corporation (USA) were used.

### Statistics and positivity criteria

For analysis of ELISPOT results for each patient an unpaired Student's t-test was applied in order to calculate significant results as compared to the internal negative (DMSO) control. To be evaluated as positive a sample (that is response to a individual or a pool of up to 40 ANDV-derived 15mer peptides) had to fulfill three criteria: (i) a significant difference (p<0.05) between sample and negative control, (ii) specific SFU had to be superior of 50/10^6^ PBMC, (iii) value had to be above a cut-off, defined as a mean + 2xSD, which was previously established in 20 healthy controls for each peptide pool.

For differences in frequencies of tetramer cell populations an unpaired Student's t-test was applied. We further studied the association of time since ANDV infection until blood sampling with the T-cell responses against N, Gn and Gc as determined by IFN-γ ELISPOT at the time point of blood sampling. A decreasing response with increasing time since infection corresponds to a loss of T-cell memory in time and is reflected by a negative correlation. We reject the null-hypothesis of no association between time and the IFN-γ-ELISPOT response at the two-sided alpha level of 0.05. Due to skewness of the response data, we log-transformed ELISPOT responses and fitted our linear regression models on the log-transformed responses and assessed the model assumptions by inspecting residual values against time.

## Supporting Information

Figure S1 Relation between HLA-B*35 expression and ANDV-specific T-cell responses. (A) Overall T-cell responses in HLA-B*35-negative or (B) HLA-B*35-positive individuals according to their clinical course of HCPS. (C) Proportion of HLA-B*35-positive individuals with significant T-cell responses towards Gn_461–475_ according to their clinical course of HCPS. Triplicates of PBMC of each patient were challenged in a 38-hour IFN-γ ELISPOT by a total of 13 pools of overlapping peptides, spanning the entire N- (aa 1–430), Gn- (aa 1–650) and Gc- (aa 641–1140) protein of Chilean ANDV. Bars indicate the sum of overall responses towards N-, Gn- and Gc-derived peptides (A, B) and towards Gn_461–475_, respectively (C).
(0.23 MB TIF)
