# Supplementary figures and images for: Highly Differentiated, Resting Gn-Specific Memory CD8+ T Cells Persist Years after Infection by Andes Hantavirus
Source: PLoS Pathog. 2010 Feb 19;6(2):e1000779. doi: 10.1371/journal.ppat.1000779 (PMC2824805; doi:10.1371/journal.ppat.1000779)

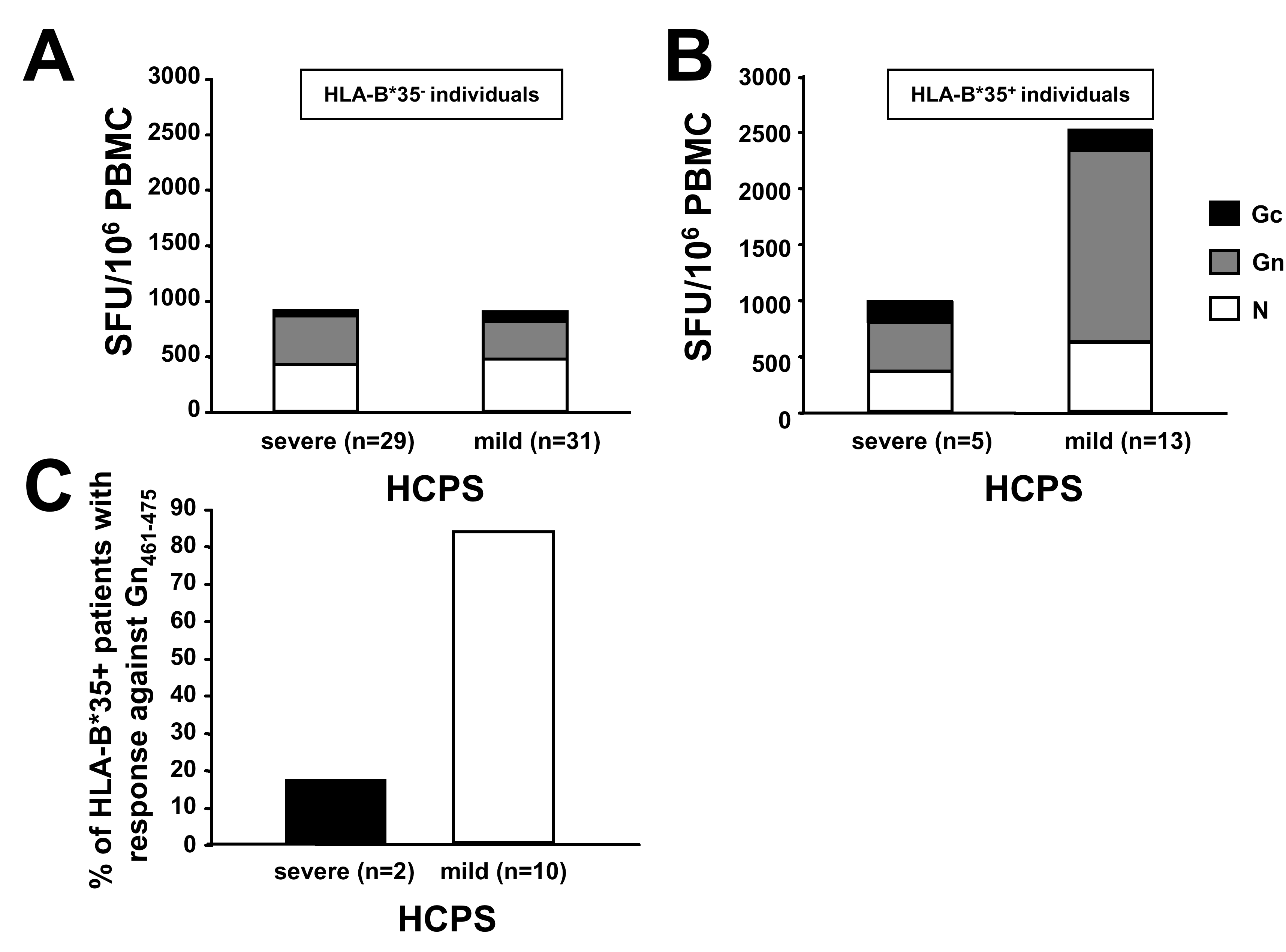

Supplement: Figure S1 — Relation between HLA-B*35 expression and ANDV-specific T-cell responses. (A) Overall T-cell responses in HLA-B*35-negative or (B) HLA-B*35-positive individuals according to their clinical course of HCPS. (C) Proportion of HLA-B*35-positive individuals with significant T-cell responses towards Gn461–475 according to their clinical course of HCPS. Triplicates of PBMC of each patient were challenged in a 38-hour IFN-γ ELISPOT by a total of 13 pools of overlapping peptides, spanning the entire N- (aa 1–430), Gn- (aa 1–650) and Gc- (aa 641–1140) protein of Chilean ANDV. Bars indicate the sum of overall responses towards N-, Gn- and Gc-derived peptides (A, B) and towards Gn461–475, respectively (C). (0.23 MB TIF) [file ppat.1000779.s001.tif]
